# Colonic medullary carcinoma: an exceedingly rare type of colorectal malignancy: a case report and review of the literature

**DOI:** 10.1186/s13256-023-04160-0

**Published:** 2023-10-18

**Authors:** Fajer Al-Ishaq, Mahmood Al-Dhaheri, Ali Toffaha, Salwa Awad, Syed Rizvi, Mohamed AbuNada, Mohamed Kurer

**Affiliations:** 1 Colorectal Surgery Unit, Hamad Medical Corporation, Doha, Qatar; 2 Laboratory and Pathology Department, Hamad Medical Corporation, Doha, Qatar

**Keywords:** Colon cancer, Medullary carcinoma, Microsatellite instability, Immunohistochemistry

## Abstract

**Background:**

Medullary carcinoma of the colon is a rare subtype of colorectal cancer that has a unique, and sometimes varied, clinical and histologic profile. It usually presents in adult patients older than 50 years. Here, we report a unique case of young male patient who initially presented with abdominal pain followed by a large bowel obstruction.

**Case presentation:**

A 40-year-old SriLankan male presented with right-sided abdominal pain and on examination, there was a palpable right iliac fossa mass. Colonoscopy and a computed tomography scan revealed cecal mass. Later, while waiting for elective resection, the patient developed symptoms and signs of a large bowel obstruction. He underwent a laparoscopic right hemicolectomy with an uneventful postoperative course. The histopathologic evaluation of the resected specimens showed invasive carcinoma with syncytial growth pattern, foci of lymphoid host response, and dirty necrosis, in keeping with a medullary carcinoma pT4a pN2b. Unlike most reported medullary carcinoma cases, this patient was young and caudal-related homeobox transcription factor 2 positive.

**Conclusion:**

We have reported another case of medullary carcinoma of the colon in a young patient with unique histologic characteristics. Reporting such cases helps in refine understanding of the histologic and genetic, as well as clinical, phenotypes of medullary carcinoma of the colon.

## Background

Colorectal cancer (CRC) is the third most common cancer globally and the second most common cause of cancer related mortality, as per the World Health Organization (WHO) factsheet (accessed 6 July 2023). The majority (around 98%) of CRCs are adenocarcinomas [1]. An exceedingly rare (0.29%) subtype is medullary [2] first described in 1999 by Jessurun [3]. Characterized initially by histologically distinct features (undifferentiated high-grade cytology, syncytial growth pattern, and prominent lymphocytic infiltration) followed by correlation with a unique molecular profile (more frequent association with microsatellite instability), it has recently been described to be more common in older individuals and females, with a better outcome when compared with poorly differentiated (usual type) adenocarcinoma [2, 4]. However, due to its rarity, prognostic data are limited and further cases with follow-up are needed to learn more about this rare subtype of adenocarcinoma. There is an increasing interest in the topic recently among clinicians and researchers, with more case reports describing the clinical and pathological features of medullary adenocarcinomas of the colon, in addition to the potential diagnostic and therapeutic approaches [5]. A recent study [7] identified overexpression of key immunoregulatory genes and features that may explain the prognostic difference from usual adenocarcinoma and provide potential therapeutic targets. In this study, we report an unusual case of medullary adenocarcinoma of the colon with some unique characteristics and summarize the reported cases in the literature.

## Case report

A 40-year-old male SriLankan presented with a 1 month history of right-sided abdominal pain, loss of appetite, weight loss, and melena, with no family history of gastrointestinal (GI) cancer. On physical examination, there was a palpable mass in the right iliac fossa (RIF) associated with tenderness.

**Fig. 1:**
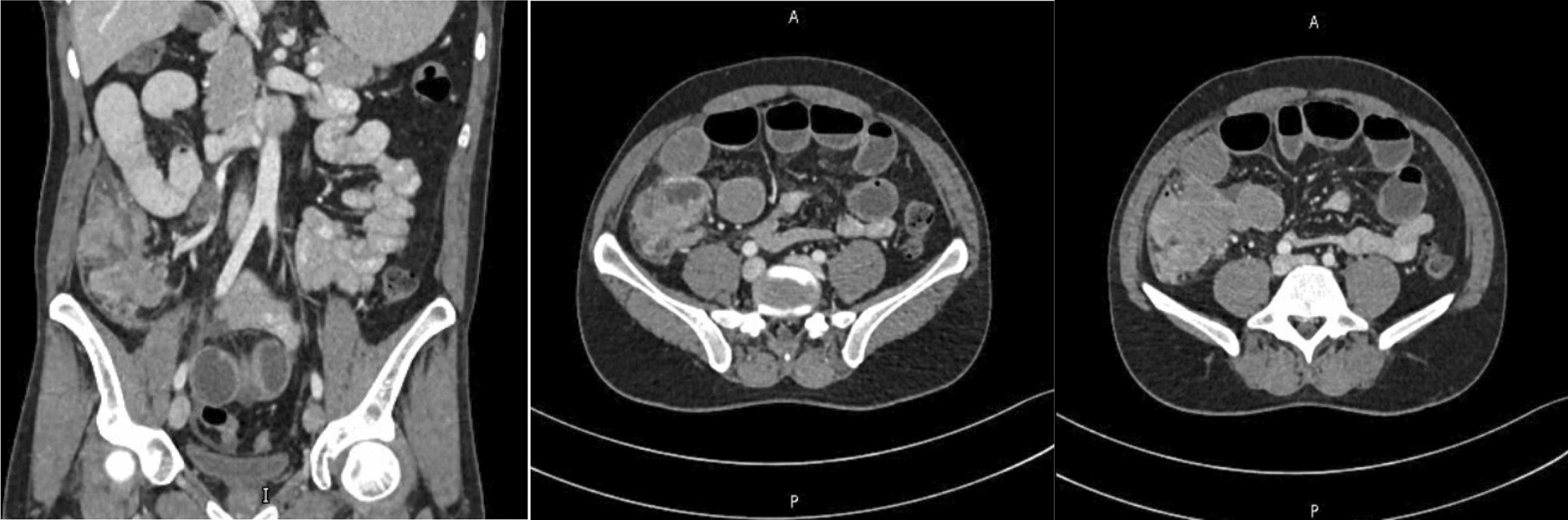
Computerized scan: mass with irregular eccentric soft tissue density involving the cecum and proximal ascending colon measuring 65 × 35 × 31 mm with multiple enlarged regional lymph nodes

**Fig. 2:**
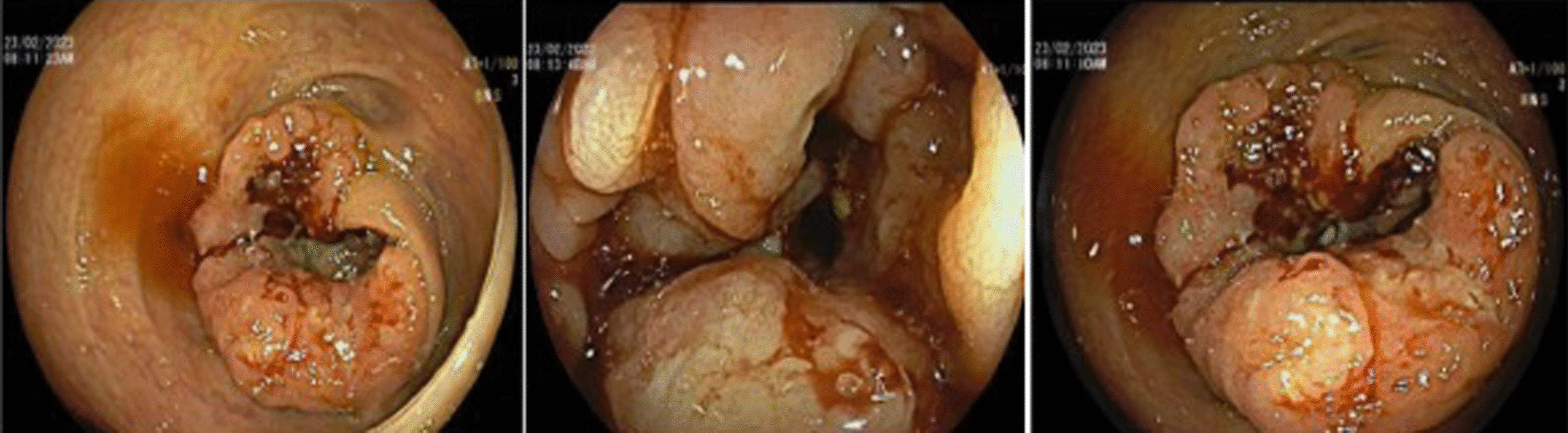
Colonoscopy: cecal, ulcero-proliferative circumferential lesion with a malignant appearance

Investigations highlighted hypochromic microcytic anemia [hemoglobin (Hgb) 7.4 mg/dL, hematocrit (Hct) 26%, mean corpuscular volume (MCV) 67 mg/dL, and mean corpuscular hemoglobin (MCH) 19.5 mg/dL]. Tumor markers were normal. Computed tomography (CT) scan of the abdomen and pelvis showed a mass with irregular eccentric soft tissue density involving the cecum and proximal ascending colon measuring 65 × 35 × 31 mm with multiple enlarged regional lymph nodes (Fig. 1). Colonoscopy showed a cecal ulcero-proliferative circumferential lesion with a malignant appearance (Fig. 2). No metastases were seen on the staging CT scan. The patient was scheduled for an elective laparoscopic right hemicolectomy; however, before his date of surgery, he presented to the emergency with an acute obstruction and was taken for emergency surgery. He underwent laparoscopic right hemicolectomy with side-to-side ileocolic anastomosis. The postoperative course was uneventful, and the patient was discharged on the seventh postoperative day.

**Fig. 3:**
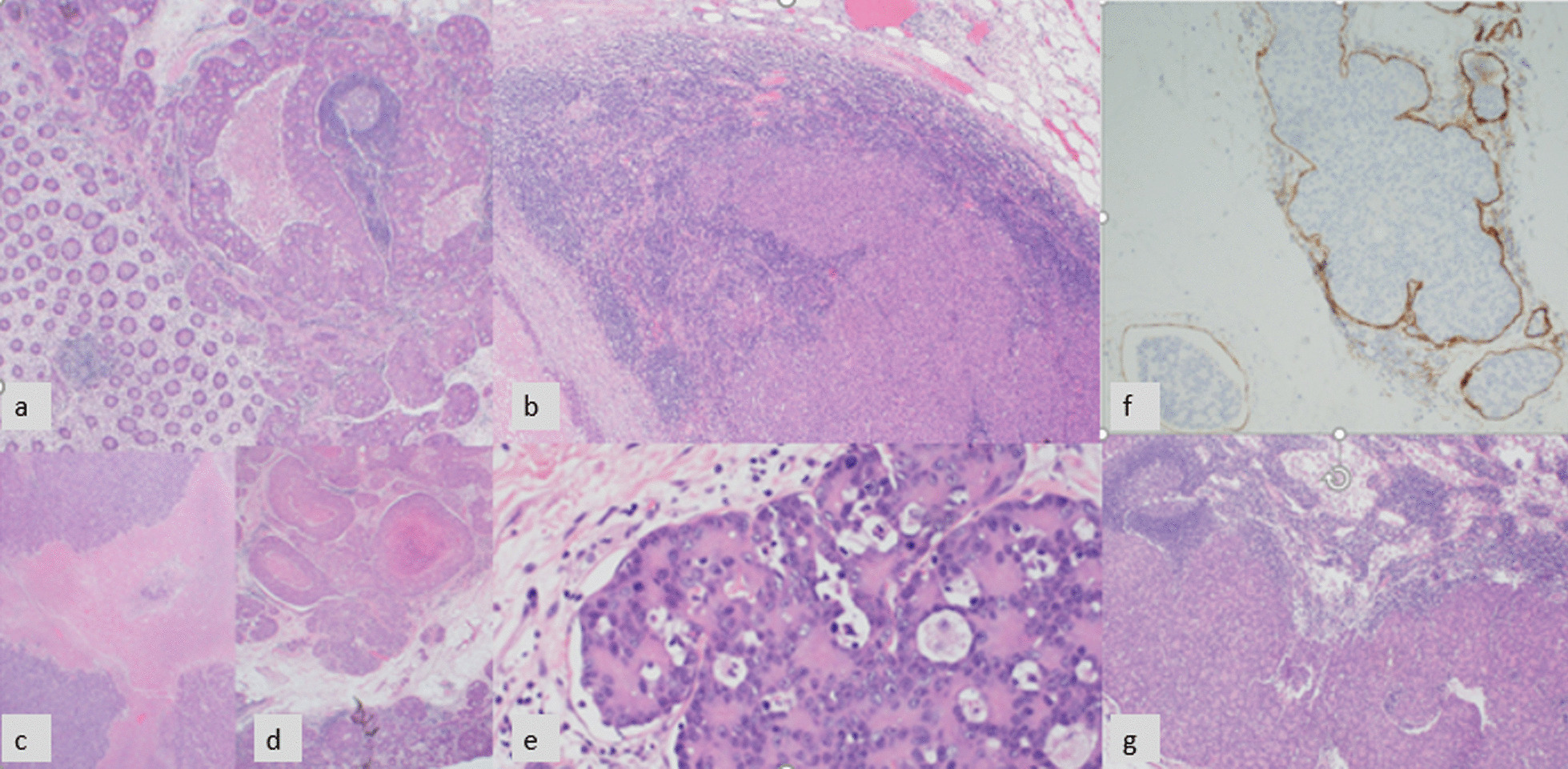
Medullary carcinoma showing prominent lymphoid infiltrate (**a**, **b**), necrosis characteristic of tumors with microsatellite instability (**c**, **d**), syncytial growth pattern with some features that resemble neuroendocrine tumors (**e**), lymphovascular invasion (**f**), and lymph node metastasis (**g**)

**Fig. 4:**
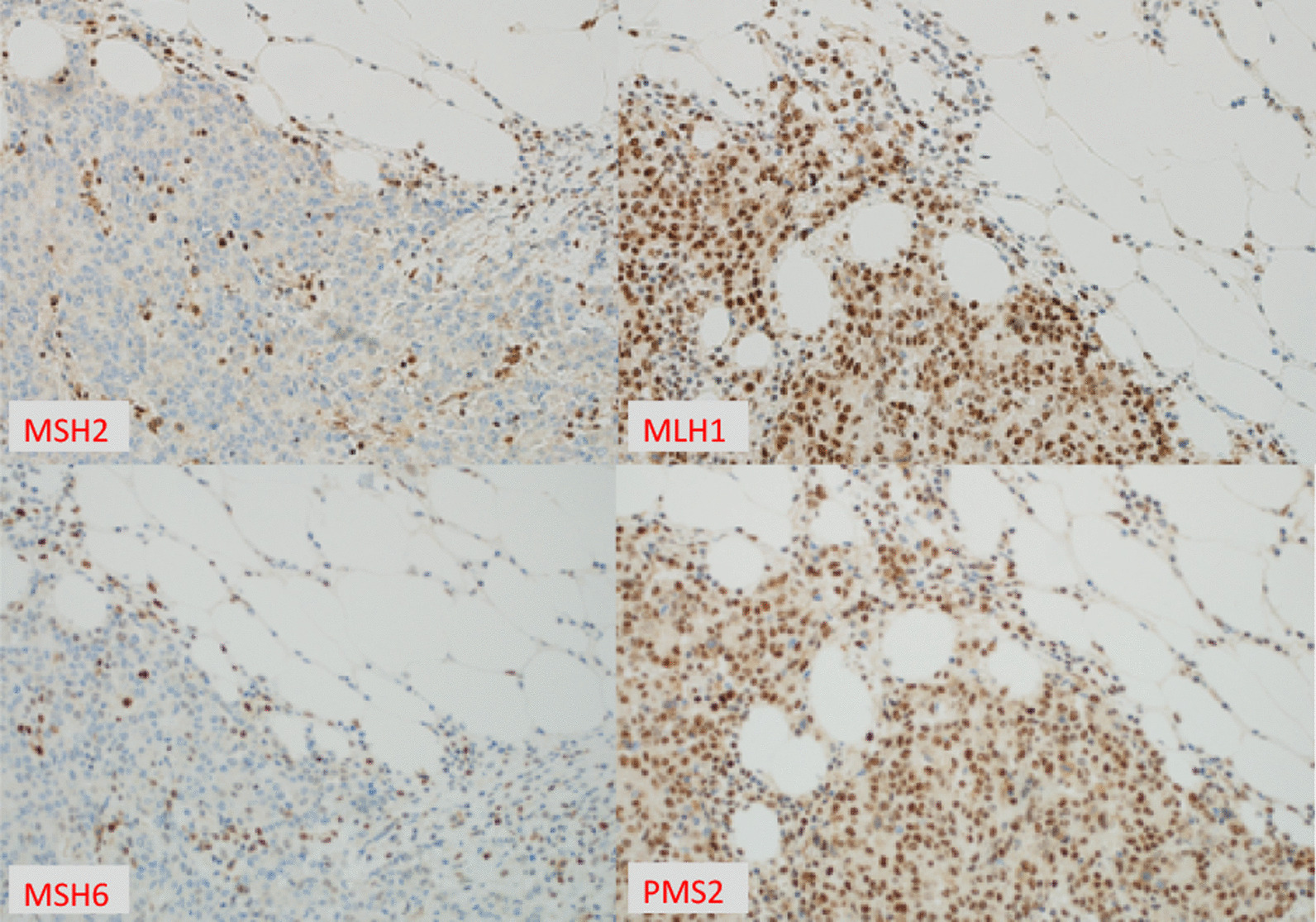
Mismatch repair status evaluation using immunohistochemical biomarkers. Loss of MSH2 (MutS homolog 2) and MSH6 (MutS homolog 6) with retained MLH1(MutL homolog 1) and PMS2 (postmeiotic segregation increased 2), confirming microsatellite instability

The histopathology evaluation of the resected specimens showed invasive carcinoma with syncytial growth pattern, foci of lymphoid host response, and dirty necrosis, in keeping with a medullary carcinoma. The tumor was present in subserosal fat (pT4a) and show metastatic carcinoma in 13 (out of 22) lymph nodes (pN2b). There was prominent lympho-vascular invasion. The margins were clear (R0) (Fig. 3). Immunohistochemical stains for mismatch repair (MMR) status showed loss of nuclear expression of both *MSH2* and *MSH6* with intact expression of *MLH1* and *PMS2* (Fig. 4). The most commonly reported loss is that of *MLH1*. The tumor cells expressed *CK20* and *SATB2* in keeping with a colorectal primary. Unlike most reported medullary carcinoma cases and like usual large bowel cancers, *caudal-related homeobox transcription factor 2 (CDX2) *was positive. Despite areas with morphological resemblance to endocrine tumor, common neuroendocrine markers (synaptophysin, chromogranin, and CD56) were negative.

## Discussion

Medullary carcinoma (MC), a relatively recently identified, extremely rare type of adenocarcinoma (with incidence reported to range from approximately 0.3% to 3%) [2, 6, 7] is characterized by a poorly differentiated carcinoma with a syncytial growth pattern, and with areas that resemble endocrine tumors but lack neuroendocrine marker expression and show a dense lymphoid host response and extensive lymphovascular invasion. So-called dirty necrosis, suggestive of microsatellite instability is also seen, with all tumors showing mismatch repair protein abnormalities. Therefore, these are best classified as a subtype of microsatellite instable carcinoma and may be sporadic or syndromic (Muir–Torre syndrome). Recently, these tumors have been shown to overexpress immunoregulatory genes [8] that, along with dense lymphoid host response, may possibly account for better overall survival when compared with ‘usual’ poorly differentiated adenocarcinoma. Colarossi *et al*. [9] highlighted immunohistochemical loss of expression of ARID1A along with a higher incidence of BRAF (V600E) mutation. Like other MSI tumors, the mutational burden of these tumors is low. This coupled with overexpression of immunoregulatory genes seems to confer a better prognosis in medullary carcinoma compared with usual-type poorly differentiated adenocarcinoma and may provide unique therapeutic targets. Therefore, correct identification and workup is essential. Especially given that the diagnosis of MC is challenging and can be confused with the diagnosis of other histological subtypes of colon MC, which necessitate a comprehensive pathological examination, in addition to immunohistochemical staining, to confirm the diagnosis of medullary adenocarcinoma of the colon [8, 10].

Table 1 summarizes the key findings in recently published cases of medullary colon cancer based on a review of recent literature on the subject. Around ten cases were found in which the MMR status, the immunohistochemical analysis of the disease, the staging, and the location of the tumor were discussed.

**Table 1:** The reported cases in the literature

Author	Year	No. of cases	Gender	Age	Presentation	Location of the tumor	Differentiation/stage	Biomarker testing	Immunohistochemical analysis
Sharma *et al*. [11]	2012	1	Female	74	Abdominal pain (LLQ), fatigue, intermittent diarrhea, and weight loss (15 lbs in 2 months)	Right-sided tumor; ascending colon	Undifferentiated	Microsatellite instability	Negative CDX-2, CK20, CK7, TTF-1, S-100 protein, MART1, PAX-8, chromogranin, synaptophysin, estrogen receptors, and hepatocyte-specific antigen
Jain *et al*. [12]	2014	1	Female	72	Bleeding per rectum Abdominal cramps and loose stool	Left-sided tumor	Poorly differentiated G3 T2N0M0	Microsatellite instability	Positive staining for MSH2 and MSH6 Loss of MLH1 and PMS2 protein expression
Cunningham *et al*. [13]	2014	2	Female	79	Weakness, abdominal pain (RLQ), anorexia, and loss of 11 kg over 1 year	Left-sided tumor; splenic flexure	Poorly differentiated G4 T3N1M1	Microsatellite instability	Positive: CDX-2, rare positive CK7 Negative: CK20, synaptophysin, and chromogranin KRAS wild type and loss of mismatch repair proteins (MLH1 and PMS2)
				81	Nausea, vomiting, diarrhea, and weight loss	Left-sided tumor; splenic flexure + distal transverse colon	Poorly differentiated G2 T4N0M0		Loss of mismatch repair proteins (MLH1 and PMS2)
Bağ *et al*. [14]	2017	1	Male	72	Abdominal pain and bleeding per rectum	Transverse colon	Moderately differentiated G2 T2N0M0	Microsatellite instability	Negative: CK20, synaptophysin negative, chromogranin, CDX2, CD56, CEAmono, calretinin Positive: p53 (20% positive+), E-cadherin, cyclin D1 (5% nuclearexpression), CD44 (80%), KI-67 (80%), and p16
Martinotti *et al*. [15]	2017	1	Female	44	Acute abdominal pain	Right-sided tumor; cecum and ascending colon	Poorly differentiated Stage 3 T3N0M0	Microsatellite instability	Positive: CK7 (focal), CAM 5.2 (focal), CKAE1/AE3 (focal), calretinin (focal), claudin 4 Negative: CK20, synaptophysin, chromogranin, CDX2, TTF-1, P63, CK5-6, CD20, CD3, CD5, CD79, MUM1, S100, ER, MART-1, EBV
Fatima *et al*. [16]	2021	1	Female	77	Lower abdominal pain, distention, vomiting, and weight loss (10 lbs)	Right-sided tumor; cecum	Poorly differentiated	Microsatellite instability	Positive: CKAE1/AE3, GATA3, calretinin, p63, and CDX2 Negative: CK7, CK20, and SATB2 Loss of PMS2 expression with intact MSH6 expression
Colarossi *et al*. [9]	2021	2	Male	70	Diffuse abdominal pain and weakness	Right-sided tumor; cecum and ascending colon	G2 T3N0M0	Microsatellite instability	Positive: calretinin and membranous beta-catenin Negative: CK20, CDX2, chromogranin, and synaptophysin Loss of MLH1, PMS, and loss of ARID1A
				62	Diffuse abdominal pain and nausea	Right-sided tumor; ileocecal	G2 T3N0M0		Positive: calretinin Negative: CDX2, CK20 and ARID1A Loss of MLH1 and PMS2 KRAS, NRAS, and BRAF were wild type
Chen *et al*. [17]	2021	1	Female	68	NA	Right-sided tumor; ascending colon	NA	Microsatellite instability	Positive: p40 and CK5/6, but negative CDX2, CK20, and SATB2 MMR deficient with loss of MLH1 and PMS2 NGS confirmed BRAF V600E mutation
Saikia [18]	2023	1	Female	77	Lower GI bleeding	Right-sided tumor; cecum	Undifferentiated G2 T3N0M0	Microsatellite instability	Positive: CDX2 Negative: CK7, CK20 synaptophysin and chromogranin, GATA 3, and PAX8 Loss of MLH1 and PMS2

*LLQ* Left Lower Quadrant, *RLQ* Right Lower Quadrant, *CDX-2* caudal-related homeobox transcription factor 2, *CK20* Cytokeratin 20, *CK7* Cytokeratin 7, *TTF-1* Thyroid transcription factor, *MART1* Melanoma-associated antigen recognized by T cells, *PAX-8* Paired-box gene 8, *MSH2* MutS Homolog 2, *MSH6* MutS homolog 6, *MLH1* MutL homolog 1, *PMS2* postmeiotic segregation increased 2, *KRAS* Kirsten rat sarcoma viral oncogene homolog, *CD56* cluster of differentiation 56, *CEAmono* carcinoembryonic antigen monoclonal antibody, *P53* protein 53, *CD44* cluster of differentiation 44, *Ki-67* Marker Of Proliferation Ki-67, *p16* protein 16, *CAM 5.2* Anti-Cytokeratin, *CK AE1/AE3* Cytokeratin AE1 / AE3, *p63* protein 63, *CK5-6* Cytokeratin 5-6, *CD20* cluster of differentiation 20, *CD3* cluster of differentiation 3, *CD5* cluster of differentiation 5, *CD79* cluster of differentiation 79, *MUM1* multiple myeloma oncogene 1, *ER* Estrogen receptor, *EBV* Epstein-Barr virus, *GATA3* GATA-binding protein 3, *SATB2* SATB Homeobox 2, *ARID1A* AT-Rich Interaction Domain 1A, *NRAS* Neuroblastoma rat sarcoma viral oncogene homolog, *BRAF* V-Raf Murine Sarcoma Viral Oncogene Homolog B, *SATB2* Special AT-rich sequence-binding protein 2, *MMR* Mismatch repair, *NGS* Next generation sequencing

Our patient was a young gentleman diagnosed with cecal adenocarcinoma and regional lymph node involvement. He had an aggressive disease that led to an obstruction that necessitated an urgent surgical resection. Unlike our patient, most cases of colon MC are in older patients, typically over 60 years old (as presented in Table 1), and predominantly in females [3]. Moreover, almost all patients who were diagnosed with colon MC presented with abdominal pain or bleeding per rectum (Table 1) rather than an obstruction.

The histopathological study of the mass showed invasive MC with features resembling neuroendocrine tumors and with only focal areas with dense lymphoid infiltrate. Like most reported cases, there was prominent lymphovascular invasion and a lack of neuroendocrine biomarkers, along with loss of MMR by immunostaining, indicating microsatellite instability. Unlike most reported cases, the tumor cells expressed CDX2 and instead of MLH1 loss, highlighted retained MLH1 and loss of both MSH2 and MSH6. BRAF was not mutated (Table 1). Hence, this case showed that MC can present with different morphological and immunohistochemical patterns and that there are variations in the ‘usual’ molecular signature. Therefore, this expands the diagnostic spectrum of MC and highlights the diagnostic challenges in the absence of an established clinical, molecular, and histopathological pattern for this type of cancer. Reporting rare cases such as colon MC helps clinicians and pathologists with the diagnosis and management of such rare conditions, and can aid in identifying the characteristic of the subtypes of colon cancer and improve our understanding of such rare entities.

## Conclusions

Medullary adenocarcinoma of the colon is an exceptionally rare subtype of colorectal cancer, with increasing knowledge about the morphology, immunophenotype and molecular profile. Recent advances in immunohistochemistry and molecular studies played a role in improving the understanding of this subtype. Still, further reports are needed for better understanding of such rare entity. It is therefore imperative that we report cases with comprehensive pathology workup, relevant clinical findings, and follow-up, to add to our understanding of this rare disease.

## Data Availability

Data are available upon request.
