# Correction: Colonic medullary carcinoma: an exceedingly rare type of colorectal malignancy: a case report and review of the literature

**DOI:** 10.1186/s13256-023-04228-x

**Published:** 2023-11-01

**Authors:** Fajer Al‑Ishaq, Mahmood Al‑Dhaheri, Ali Toffaha, Salwa Awad, Syed Rizvi, Mohamed AbuNada, Mohamed Kurer

**Affiliations:** 1 Colorectal Surgery Unit, Hamad Medical Corporation, Doha, Qatar; 2 Laboratory and Pathology Department, Hamad Medical Corporation, Doha, Qatar

**Correction: Journal of Medical Case Reports (2023) 17:434** 10.1186/s13256-023-04160-0

Following publication of the original article [1], an error was identified in the Funding section which incorrectly stated there was no funding.

The section currently reads:

**Funding**

No fund received for this article.

The section should read:

**Funding**

Funding Open Access funding provided by the Qatar National Library.

The funding section has been updated above and the original article [1] has been corrected.
